# Multimodal imaging–guided awake transnasal fiberoptic tracheal intubation in a patient with giant palatal pleomorphic adenoma: a case report

**DOI:** 10.3389/fonc.2025.1723032

**Published:** 2025-12-03

**Authors:** Feng-Jiao Zhang, Chao Zhou

**Affiliations:** Department of Anesthesiology, The Fourth Hospital of Hebei Medical University, Shijiazhuang, Hebei, China

**Keywords:** palatal neoplasms, pleomorphic adenoma, bronchoscopy-guided, awake intubation, multimodal imaging

## Abstract

We report the case of an 80-year-old male with a giant palatal pleomorphic adenoma (7.0 × 5.0 × 5.5 cm) that caused progressive dyspnea, dysphagia, and oropharyngeal airway distortion after 40 years of indolent growth. To our knowledge, this represents one of the largest documented cases in which awake transnasal tracheal intubation was performed for an intraoral tumor. Preoperative evaluation revealed nasopharyngeal-nasal junction narrowing, a mouth opening of 3 cm, Mallampati grade IV, and preserved glottic function. Multimodal imaging—including MRI, 3D CT reconstruction, and fiberoptic laryngoscopy—was critical in delineating tumor extent and airway anatomy, guiding a tailored airway management strategy. Awake transnasal fiberoptic tracheal intubation was performed via the right nostril using dexmedetomidine sedation, topical and regional anesthesia, and ultrasound-guided superior laryngeal nerve blocks. Intubation was successful on the first attempt, without the need for tracheotomy or procedural complications. The procedure lasted 90 minutes, with minimal blood loss and stable hemodynamics. The patient was extubated on postoperative day 1, discharged on day 10, and remained clinically stable at 6-month follow-up.

## 1 Introduction

Salivary gland tumors are among the most common types of oral and maxillofacial tumors, accounting for approximately 2.3% of all such lesions (1). Among various salivary gland tumors, pleomorphic adenomas—also referred to as benign mixed tumors—are the most clinically prevalent. Although typically benign, pleomorphic adenomas possess distinct histopathological and growth characteristics. Most arise from the parotid gland, although they may also originate from minor salivary glands, such as the palatal, submandibular, and sublingual glands (2, 3). Among patients presenting with extensive intraoral tumors, pleomorphic adenomas of palatal origin warrant particular attention due to their anatomical location. Such tumors often grow protrusively into the oral cavity, with potential invasion of both hard and soft palatal structures and extension into the oropharyngeal isthmus, resulting in substantial space-occupying lesions. Their firm consistency, hypervascularity, and potential for osseous invasion of the palatal bone contribute to distortion of the palate’s anatomical structure. These characteristics not only determine the diagnostic and therapeutic approach but also pose significant challenges for airway management during subsequent surgery.

The establishment of a safe and reliable airway by anesthesiologists is a prerequisite for surgical resection of palatal pleomorphic adenomas to ensure the smooth progress of surgery and patient safety. However, the tumor’s anatomic location and growth may complicate tracheal intubation. The primary challenges are as follows. (i) Airway obstruction and compromised passage: the tumor’s protrusion into the oral cavity compresses the hard and soft palates, potentially causing the soft palate to sag and block the oropharyngeal isthmus. This anatomical narrowing impedes smooth advancement of the endotracheal tube into the pharynx through the oral route and may result in impaction, increasing the likelihood of intubation failure. (ii) Impaired glottic visualization: The tumor may push the tongue inferiorly and posteriorly, reducing the operating space between the tongue and palate. During laryngoscopy, the tumor or displaced tongue may obstruct the view of the glottis, thereby diminishing the success rate of intubation and increasing the risk of an emergency airway. (iii) Risk of hemorrhage and asphyxia: Given the tumor’s rich vascularity, inadvertent contact with the laryngoscope or endotracheal tube may precipitate rupture and hemorrhage. Blood entering the airway further obscures visualization and may lead to airway obstruction and asphyxiation. Tumors involving the soft palate pose an increased risk of posterior pharyngeal bleeding, exacerbating airway compromise.

To address these challenges, anesthesiologists need to adopt targeted strategies: perform accurate evaluation of the tumor’s location, size, and spatial relationship with the airway through multimodal imaging examinations such as MRI and 3D CT reconstruction before surgery; formulate an individualized tracheal intubation plan based on the specific airway conditions to prevent loss of airway control following induction of general anesthesia; and engage in multidisciplinary collaboration with surgical teams to jointly develop contingency plans to ensure the safety and controllability of the airway management process. This case report exemplifies the successful application of these principles in securing a safe airway in a patient with a giant palatal pleomorphic adenoma, offering a valuable reference for similar clinical scenarios.

## 2 Case report

### 2.1 Case summary

The patient, an 80-year-old man, 162 cm in height, and weighing 53 kg, presented with a palatal mass of over 40 years’ duration and progressive dyspnea for more than 1 month. The patient first noticed the palatal tumor over 40 years ago but did not pay much attention to it. One year prior to presentation, the patient noticed the tumor’s accelerated growth and self-administered traditional Chinese medicine orally without improvement. In the month preceding admission, he developed positional dyspnea, was unable to lie supine, occasionally woke up at night due to suffocation, and had difficulty eating and speaking. These symptoms progressively worsened, prompting him to seek medical care at our hospital.

The patient had a 20-year history of hypertension, managed with amlodipine besylate (2.5 mg once daily), maintaining blood pressure within 120–130/75–80 mmHg (1 mmHg = 0.133 kPa). He had no history of diabetes mellitus or coronary artery disease. He had a 60-year history of smoking, consuming approximately 20 cigarettes daily, and denied alcohol consumption.

Facial symmetry and mouth opening were within normal limits. Intraoral examination revealed a bulge on the palatal mucosa, measuring approximately 7.0 cm × 5.0 cm × 5.5 cm, with a central spherical protrusion, compressing the tongue. The lesion extended bilaterally to the maxillary tuberosities, with an indistinct and nonpalpable posterior margin. The mass was moderately firm, non-tender, and poorly demarcated. Visualization of the oropharynx was not possible. No obvious redness, swelling, or abnormal exudate was noted from the salivary duct orifices. Cervical lymphadenopathy was absent. Systemic examination revealed no additional abnormalities.

**Figure 1:**
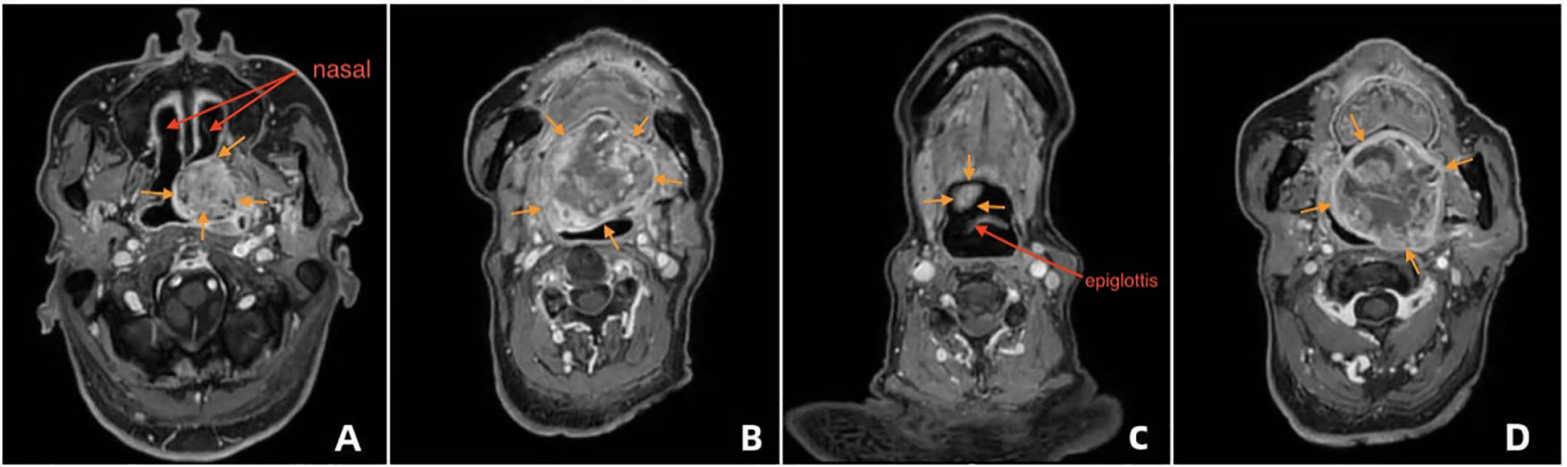
Preoperative imaging of the palatal mass and airway distortion **(A)** MRI showing the soft-tissue mass along the left nasopharyngeal wall protruding into the nasal cavity with heterogeneous signal and contrast enhancement. **(B)** MRI sagittal view illustrating the mass extending downward toward the oropharynx and near the epiglottic area. **(C)** MRI showing preserved glottic structures with no thickening of the aryepiglottic folds. **(D)** CT scan illustrating the mass boundary, calcifications at the edge, and the inferior extent toward the epiglottic region, with marked narrowing of the oropharyngeal lumen at the C1 level.

No obvious abnormalities were found in laboratory examinations. Electrocardiography revealed sinus rhythm, left axis deviation, and Q waves in leads V2–V3. Echocardiography revealed degenerative changes of the aortic valve and reduced left ventricular diastolic function. Head and neck MRI showed a soft tissue mass along the left nasopharyngeal wall, exhibiting mixed signal intensities on T1-weighted imaging (T1WI) and T2-weighted imaging (T2WI). T1WI showed mildly hyperintense signals, and T2WI revealed multiple nodular hyperintense foci with smooth margins. Contrast-enhanced scanning showed heterogeneous enhancement of septations and necrotic areas. The mass originated from the left wall of the nasopharynx, extended anteriorly into the nasal cavity (**Figure 1A**), obliterated the left pharyngeal recess, and descended anteroinferiorly toward the oropharynx (**Figure 1B**), with its inferior pole reaching the epiglottic apex. No thickening of the aryepiglottic folds was noted (**Figure 1C**). The mass measured approximately 7.0 × 5.3 × 5.0 cm (vertical × longitudinal × transverse dimensions), causing significant oropharyngeal narrowing. At the level of the first cervical vertebra (C1), the lesion abutted the posterior oropharyngeal wall, nearly occluding the oropharyngeal lumen (**Figure 1D**). Skull CT revealed a soft tissue mass on the left palate with indistinct margins protruding into the posterior nasal cavity. The left pharyngeal recess was shallow, and the lesion extended inferiorly and rightward toward the epiglottis, with peripheral calcifications. Contrast-enhanced CT showed mild heterogeneous enhancement, with a maximal longitudinal diameter of approximately 5.8 cm.

The provisional diagnosis at admission was palatal neoplasm and hypertension.

Regarding preoperative risk assessment, the patient was classified as American Society of Anesthesiologists (ASA) Physical Status Grade IV and New York Heart Association (NYHA) Cardiac Function Class I. He was capable of performing basic household tasks, corresponding to a metabolic equivalent of 4 METs. Pre-anesthetic airway evaluation revealed a mouth opening of 3 cm, modified Mallampati Grade IV, mentohyoid distance of 6.0 cm, normal cervical spine mobility, and limited tumor mobility.

### 2.2 Anesthesia and surgical conditions

**Figure 2:**
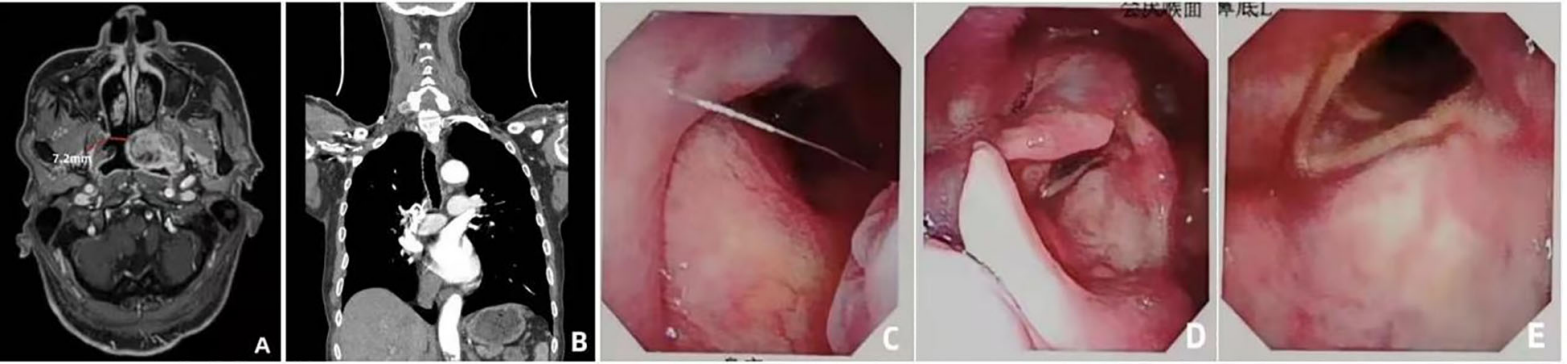
Endoscopic evaluation and airway assessment **(A)** Coronal CT showing stenosis at the right nasal-nasopharyngeal junction (narrowest diameter: 7.2 mm). **(B)** Sagittal CT showing normal structure of the larynx and lower airway. **(C)** Transnasal fiberoptic laryngoscopy showing a smooth-surfaced neoplastic bulge in the soft palate area. **(D)** Fiberoptic view of small smooth protrusions on the laryngeal surface of the epiglottis. **(E)** Fiberoptic view of symmetrical bilateral vocal cords with normal closure function.

On imaging evaluation (MRI + CT), the right nasal cavity was patent, although the junction between the right nasal cavity and the nasopharynx was partially obstructed by the tumor, with the narrowest segment measuring approximately 7.2 mm (**Figure 2A**). No significant compression was noted in the laryngeal or epiglottic regions, and the anatomical integrity of the middle and lower airway segments was maintained (**Figure 2B**).

On endoscopic evaluation (transnasal fiberoptic laryngoscopy), the nasopharyngeal mucosa appeared intact. A neoplastic protrusion was visualized in the soft palate region, with a smooth surface contour (**Figure 2C**). Small, smooth elevations were noted on the laryngeal surface of the epiglottis, which maintained normal mobility (**Figure 2D**). No abnormal masses were observed on the bilateral vocal folds, which demonstrated symmetrical motion and complete glottic closure (**Figure 2E**). The hypopharynx and glottic structures exhibited normal functional parameters.

Based on these findings—specifically, the localized stenosis at the nasal cavity–nasopharynx junction and the absence of obstruction in the laryngeal and glottic regions— a fiberoptic bronchoscope–guided awake transnasal tracheal intubation was selected as the primary airway management strategy to mitigate the risk of airway compromise during the induction of general anesthesia.

The right nasal cavity was chosen due to its unobstructed passage, thereby circumventing the narrowed segment.

Real-time visualized guidance by a fiberoptic bronchoscope was employed to ensure accurate intubation through the narrowed anatomical corridor and avoid tumor injury. A full set of tracheotomy instruments and supplies was prepared before surgery. In case of the following emergency situations—such as failed intubation, acute airway obstruction or hemorrhage during intubation, or persistent inability to maintain blood oxygen saturation—a tracheotomy would be performed immediately to establish a secure artificial airway and maintain adequate ventilation.

Upon arrival in the operating theater, peripheral intravenous access was secured, and isotonic saline infusion was initiated. Electrocardiographic monitoring revealed oxygen saturation (SpO_2_) of 98%, heart rate (HR) of 77 bpm, and invasive arterial blood pressure of 154/65 mmHg via the left radial artery. Supplemental oxygen was administered via face mask at flow rate of 5 L/min; next, 0.5 mg penehyclidine hydrochloride was administered to reduce airway secretions. Dexmedetomidine was continuously infused at a dose of 1 μg/kg over 10 minutes to achieve conscious sedation. Cotton swabs moistened with 2% lidocaine were used for surface anesthesia of the right nostril, concurrently allowing assessment of nasal patency. Oropharyngeal mucosa was similarly anesthetized. An ultrasound-guided bilateral superior laryngeal nerve block was administered (3 ml of 2% lidocaine on each side) (4). Although spraying lidocaine through the working channel of a fiberoptic bronchoscope is considered a less invasive approach than cricothyroid membrane puncture, fiberoptic bronchoscopes equipped with such channels typically have a relatively larger outer diameter. Given that the narrowest segment of the patient’s airway measured only 7.2 mm, we were concerned that a thicker bronchoscope might cause airway bleeding. Thus, we opted for cricothyroid membrane puncture instead, spraying 3 ml of 2% lidocaine to effectively anesthetize the tracheal mucosa and ensure patient comfort during intubation. A fiberoptic bronchoscope was inserted through the right nostril and advanced through the nasal cavity. The angle of the endoscope body was adjusted continuously and it was slowly advanced along the right edge of the tumor, allowing visualization of the pharyngeal structures. During the inspiratory phase, the glottis was clearly visualized. The patient was instructed to inhale, facilitating endoscopic advancement into the trachea. Upon identification of the tracheal rings and carina, a 6.0# reinforced wire endotracheal tube was inserted, and its placement was reconfirmed. The patient remained cooperative, calm, and exhibited no significant coughing or movement. After successful intubation, intravenous administration of remimazolam (10 mg), sufentanil (20 μg), and cisatracurium (12 mg) was performed. The endotracheal tube was secured and connected to the anesthesia workstation for mechanical ventilation.

The operation lasted for 90 minutes. Estimated intraoperative blood loss was 10 ml, and total fluid administration amounted to 900 ml.

**Figure 3:**
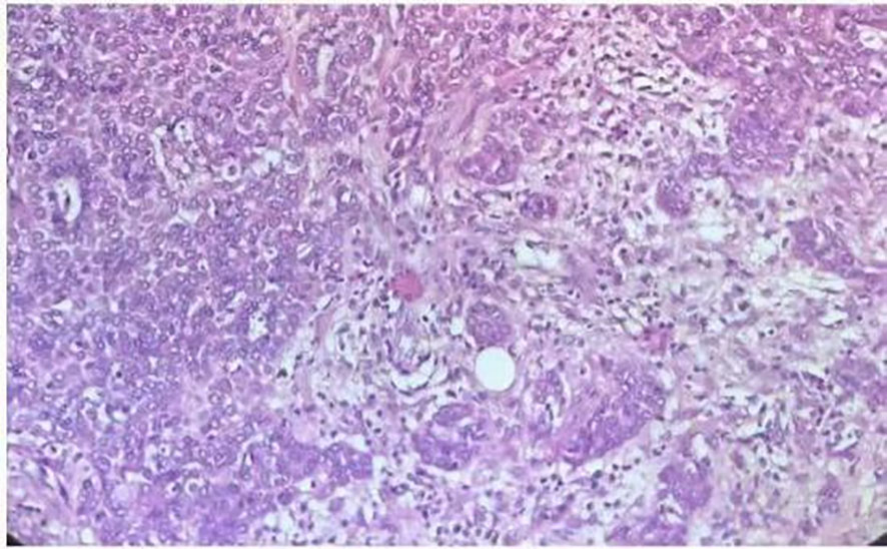
Hematoxylin and eosin-stained(×200)section showing features of pleomorphic adenoma with invasive growth characteristics.

Postoperatively, 5 ml of 2% lidocaine was sprayed into the endotracheal tube to provide sufficient surface anesthesia and improve patient tolerance. The patient was transferred to the post-anesthesia care unit (PACU); he regained consciousness 10 minutes later and was transferred to the ward with the endotracheal tube after 1 hour of observation. Oxygen supplementation was continued via the tube at 2 L/min. Vital signs were as follows: SpO2 99%, blood pressure 152/78 mmHg, HR 82 beats/min, and body temperature 37.0°C. On postoperative day 1, the endotracheal tube was extubated. On postoperative day 2, the patient exhibited no hoarseness, sore throat, or speech impairment. Histopathological analysis confirmed a pleomorphic adenoma exhibiting invasive growth and focal malignant transformation consistent with myoepithelial carcinoma (**Figure 3**). Immunohistochemical staining revealed the following profile: AE1/AE3 (+), Vimentin (+/-), CK5/6 (+), P63 (+), GFTP (-/+), Calponin (+), S100 (focal +), Ki67 (positive cells accounting for 15% in high-expression areas), CD117 (+/-), CK7 (+). All clinically submitted margin specimens, including intratumoral marginal tissue, extratumoral marginal tissue, superior marginal tissue of the tumor, and inferior marginal tissue of the tumor, were negative for tumor involvement. The patient was successfully discharged on postoperative day 10. Telephone follow-up at 6 months post-surgery indicated stable clinical status. The timeline of the case report is shown in **Table 1**.

**Table 1:** Case report timeline.

Flowchart	Timeline
Onset and symptoms	40 years of palatal mass, progressive dyspnea for 1 month, dysphagia, dysarthria
	ASA IV risk factors: elderly (80 years), hypertension, long smoking history
Preoperative assessment	Imaging: MRI and CT show left nasopharyngeal mass extending to oropharynx; oropharyngeal lumen narrowed at C1 level; palatal mass approx. 7.0 x 5.0 x 5.5 cm
	Airway assessment: mouth opening ~3 cm, modified Mallampati IV, mentohyoid distance 6.0 cm; larynx/glottis function preserved
	Plan: multimodal imaging guiding awake transnasal fiberoptic intubation; backup tracheotomy if needed
Day of surgery: anesthesia planning and preparation	Right nasal cavity chosen for intubation (patent; narrows at nasal–nasopharyngeal junction ~7.2 mm)
	Endoscopic evaluation: soft palate tumor bulge; laryngeal epiglottic surface mild protrusions; normal glottic function
	Emergency readiness: full tracheotomy kit prepared; emergency plan defined
Anesthesia induction and airway management	Sedation: dexmedetomidine 1 μg/kg over 10 minutes
	Local/topical anesthesia: 2% lidocaine for nasal/oropharyngeal surfaces; ultrasound-guided bilateral superior laryngeal nerve blocks (3 mL 2% lidocaine each side)
	Airway technique: fiberoptic bronchoscope through right nasal cavity; glottis visualized during inspiratory phase
	Tracheal intubation: 6.0 mm reinforced endotracheal tube; successful on first attempt
	Adjuncts: remimazolam 10 mg, sufentanil 20 μg, cisatracurium 12 mg given after intubation
	Intraoperative data: stable hemodynamics (BP ~148–156/62–68 mmHg)
Surgical procedure	Duration: 90 minutes
	Blood loss: 10 mL
	Fluids: 900 mL total
Immediate postoperative course	Postoperative management: local lidocaine on endotracheal tube for surface anesthesia
	Recovery: awake, SpO2 ~99% in PACU; extubated on postoperative day 1
	Oxygen: nasal cannula or tracheal tube oxygen at 2 L/min as needed
Postoperative pathology and recovery	Pathology: pleomorphic adenoma with regional malignant transformation (myoepithelial carcinoma)
	Postoperative status: discharged day 10
	Follow-up: telephone check at 6 months, patient in good condition

## 3 Discussion

Airway management complexity in this case was attributable to the convergence of multiple high-risk factors: anatomical obstruction from the tumor, comorbid physiological vulnerabilities, and potential risks from tumor biological characteristics. The palatal pleomorphic adenoma measured 7.0 × 5.0 × 5.5 cm, representing one of the largest intraoral primary tumor of its kind documented to date. The mass exhibited a spherical protrusion into the oral cavity, compressing the tongue, and extended inferiorly to the epiglottic apex, nearly obliterating the oropharyngeal lumen at the C1 vertebral level. This anatomical distortion directly led to two key problems: (i) complete loss of the transoral access due to the absence of operating space (modified Mallampati Grade IV, unable to expose the glottis); and (ii) risk of uncontrollable airway after general anesthesia induction (soft palate sagging and tumor-induced obstruction after muscle relaxation, leading to asphyxia).

Additionally, the patient’s advanced age, long-standing hypertension and smoking history compounded the risk. Electrocardiography showed Q waves in leads V2–V3, and echocardiography showed reduced left ventricular diastolic function with ASA Grade IV, indicating poor tolerance to hypoxia. Any desaturation during intubation—whether due to hemorrhage-induced visual obstruction or mechanical airway blockage—could rapidly precipitate cardiovascular or cerebrovascular events, significantly narrowing the margin for error. Although histologically benign, the tumor’s hypervascularity and focal malignant transformation introduced additional risks. Contact between airway instruments and the tumor could provoke uncontrollable bleeding. The blood flowing into the already narrow oropharyngeal cavity could lead to visual field loss + airway obstruction. Through meticulous preoperative planning, our team successfully performed tracheal intubation, offering a valuable reference for similar cases.

Preoperative planning for this case involved a 2-hour multidisciplinary team (MDT) meeting involving anesthesiologists, otolaryngologist, radiologists, and intensivists. Radiologists interpreted imaging to delineate the tumor-airway relationships; surgeons confirmed tumor resectability and formulated contingency plans for intra operative bleeding; anesthesiologists presented the proposed airway management strategy.

To inform our anesthesia plan with prior clinical experience, we systematically reviewed six published cases of giant palatal and parapharyngeal pleomorphic adenomas (maximum tumor diameter > 5 cm) reported between 2010 and 2024, focusing on airway management strategies and outcomes (**Table 2**) (5–10). Among them, four cases (66.7%) required elective or emergency tracheotomy to secure perioperative ventilation due to airway compromise from tumor compression or obstruction; one case achieved successful intubation via right nasal fiberoptic laryngoscopy without tracheotomy (5); while another case initially failed direct laryngoscopy intubation (Cormack-Lehane Grade 3), succeeded with video laryngoscope assistance, and subsequently underwent delayed tracheotomy for rehabilitation (7).

**Table 2:** Summary of the six previously published cases of giant palatal and parapharyngeal pleomorphic adenomas.

Author (Year)	Tumor size (cm)	Airway management strategy	Complications	Outcome
Nnko et al. (2023) (5)	9.4×7.9×8.3	Fiber-optic guided endotracheal intubation via the right nostril combined with general anesthesia	None	The tumor was completely resected. No recurrence was observed at 6 and 12 months postoperatively, and swallowing and speech functions were not affected.
Luo and Liu (2014) (6)	5.0×4.5×4.0	Tracheostomy under local anesthesia, followed by endotracheal intubation under general anesthesia	None	The tracheostomy tube and sutures were removed at 1 week postoperatively; the incision healed, and no recurrence was noted at 8 months postoperatively.
Ono et al. (2022) (7)	5.4×5.0×3.5	Video laryngoscope-assisted endotracheal intubation (initial direct laryngoscopy failed) + subsequent tracheostomy	Cardiopulmonary arrest induced by upper respiratory tract infection (spontaneous circulation restored after rescue)	Patient was transferred to a rehabilitation hospital without neurological sequelae; tumor resection was postponed until functional improvement.
Bist et al. (2017) (8)	10×8.2×5.8	Emergency tracheostomy (unable to maintain oxygen saturation due to acute airway obstruction)	None	The tumor was completely resected via a transcervical approach. The tracheostomy site was closed at 2 weeks postoperatively, and no recurrence was found during 1-year follow-up.
Bordoy-Soto et al. (2016) (9)	9×9×10	Tracheostomy was performed under local anesthesia + sedation (conventional intubation impossible), followed by surgery under general anesthesia	Postoperative oronasal fistula (approximately 2 cm in diameter)	The tumor was completely resected; no recurrence was observed at 10 months of follow-up; local flap repair for the fistula and subsequent rehabilitation were planned.
Arab et al. (2014) (10)	Not specified (huge craniofacial tumor)	Sedation with dexmedetomidine + ketamine administered via gastrostomy tube + EMLA cream application at the planned tracheostomy site, followed by tracheostomy under local anesthesia	None	The operation went smoothly; the patient could open eyes on command, had no recall of the operation, and expressed high satisfaction.

In contrast our case benefited from a multimodal preoperative evaluation that enabled successful fiberoptic-guided transnasal intubation, thereby avoiding complications related to tracheotomy, including neck infection, tracheal stenosis, and postoperative dysphagia. This approach underscores the value of preoperative refined anatomical evaluation in minimizing invasive airway interventions. Our stratgy is consistent with the 2022 American Society of Anesthesiologists Practice Guidelines for Management of Difficult Airway, which advocate for detailed anatomical imaging to guide airway planning and reduce procedural risk (11, 12).

It is noteworthy that one of the previously reported six cases encountered challenges related to intubation technology (7): poor glottic exposure with direct laryngoscopy due to the tumor occupying the oropharyngeal space—not due to awake intubation failure or tumor bleeding. In the case reported by Nnko et al., successful transnasal fiberoptic intubation was achieved following through preoperative evaluation, with no evidence of nasopharyngeal stenosis (5). In the case reported by Arab et al., awake fiberoptic intubation was considered, but ultimately not performed due to patient non-cooperation (10).

These findings suggest that the success rate of airway procedures highly depends on accurate preoperative understanding of the tumor location and airway anatomy. Our implemented strategy avoided such risks through targeted multimodal evaluation: CT provided high-resolution spatial data, allowing accurate location and measurement of a 7.2-mm stenotic segment at the right nasal cavity-nasopharynx junction, which aided us in selecting the correct tube size. MRI offered superior soft tissue contrast, identifying an avascular area at the right margin of the tumor (confirmed by low signal on T2-weighted imaging), minimizing the risk of procedure-related bleeding. Transnasal fiberoptic laryngoscopy enabled dynamic, real-time visualization of glottic mobility and airway patency.

A key insight from this case is that multimodal imaging forms the foundation for precise airway management. Conventional assessments—such as mouth opening, mentohyoid distance, and Mallampati classification—offer only superficial insights. In contrast, the integrated use of MRI, 3D CT reconstruction, and fiberoptic laryngoscopy enables a comprehensive three-dimensional mapping of the airway, facilitating a safe and minimally invasive airway strategy. The advantages and disadvantages of different imaging examinations in airway evaluation are detailed in **Table 3**. Collectively, the experience from previous literature and the success of this case indicate that awake intubation is not universally safe or applicable. Its success depends fundamentally on comprehensive and objective preoperative evaluation of airway anatomy.

**Table 3:** Multimodal airway evaluation and decision-support in awake intubation for giant palatal tumor.

Evaluation method	Advantages and disadvantages of different evaluation methods	Decision-support value
Head, Neck, and Chest CT	1. Identified tumor calcification (peripheral calcified margins) and excluded palatal bone invasion; 2. Localized airway stenosis to the junction of the right nasal cavity and nasopharynx (the narrowest part was 7.2 mm); confirmed normal anatomy of the larynx and trachea. 3.CT cannot assess soft-tissue vascularity.	Ruled out “lower airway obstruction”; supported feasibility of transnasal ventilation; delineated the stenotic segment and guided selection of the intubation route, avoiding the more constricted left side.
Head and neck MRI	1. Defined tumor soft tissue boundaries and its spatial relationship with the anterior and posterior walls of the oropharynx and epiglottis); 2. Confirmed that the tumor’s inferior margin reached only the epiglottis apex without compressing the larynx or glottis; 3. Identified internal necrosis to avoid trauma-induced bleeding during intubation. 4. MRI overestimates airway stenosis in edematous tissues.	Ruled out “laryngeal obstruction risk”; provided key evidence supporting “awake intubation without tracheotomy”; mapped safe tumor margins (looser tissue on the right), guiding fiberoptic bronchoscope advancement.
Transnasal fiberoptic laryngoscopy	1. Enabled real-time observation of the patency of the nasal cavity-nasopharynx (the right nasal mucosa was smooth without obvious stenosis); 2. Enabled direct visualization of the tumor bulge in the soft palate (smooth surface without ulceration); 3. Confirmed glottic mobility (bilateral vocal cords were symmetrical with good closure). 4. Transnasal fiberoptic laryngoscopy does not allow for the observation of the conditions inside the patient’s trachea.	Verified feasibility of the right nasal route; excluded “glottic dysfunction” to ensure ventilation efficiency after intubation; enabled real-time evaluation of the tumor surface to minimize the risk of operational injury.

Based on the airway anatomy and pathology in this case, three potential airway management strategies were evaluated. The rationale for selecting awake transnasal fiberoptic bronchoscope intubation is detailed below:

Intubation after transoral general anesthesia induction: This approach was categorically contraindicated. The patient exhibited a modified Mallampati Grade IV classification, with the oropharynx completely invisible and the tumor occupying over 90% of the palatal vault. These anatomical constraints rendered laryngoscope insertion infeasible. Furthermore, neuromuscular blockade following induction would result in soft palate collapse, exacerbating oropharyngeal obstruction and precipitating post-induction asphyxia (13)—a scenario incompatible with the safety requirements for ASA Grade IV patients.

Prophylactic tracheotomy: Although tracheotomy offers definitive airway access, its complication rate ranges from 4.3% to 40% (14). Specific risks include hemorrhage (2%–4%), mucus plug formation (2.7%) (15), and long-term tracheal stenosis (approximately 8.8%) (16). Given the patient’s advanced age (80 years), these complications posed significant morbidity and potential mortality. Additionally, prolonged postoperative tube dependence contradicts the goal of “minimally invasive intervention and rapid rehabilitation.” Tracheotomy was reserved as an emergency contingency in the event of failed awake intubation or acute hemorrhagic airway compromise.

Awake transnasal fiberoptic bronchoscope intubation: This technique offers the dual guarantee of “visualization and procedural control.” The fiberoptic bronchoscope can observe the intubation path in real-time and advance slowly along the tumor’s right edge (MRI indicated no obvious vascular plexus in this area), thereby minimizing hemorrhagic risk. Simultaneously, maximal glottis opening during the inspiratory phase can improve intubation success rates. In this case, intubation was successful on the first attempt.

Dexmedetomidine was administered to maintain awake sedation in this 80-year-old high-risk patient (ASA Grade IV) with hypertension and a giant palatal pleomorphic adenoma. As a highly selective α_2_-adrenergic receptor agonist, dexmedetomidine modulates sedation, preserves spontaneous breathing, and promotes patient cooperation by activating the α_2_ receptors in the locus coeruleus of the central nervous system and α_2_ receptors in the spinal cord. A dosage of 1 μg/kg infused over 10 minutes was selected based on recent evidence supporting its efficacy and safety; this regimen reliably achieves Ramsay Sedation Scale Grade 2, offering a controllable depth of sedation without excessive respiratory or cardiovascular depression (17). The patient remained responsive to verbal commands throughout the procedure, minimizing movement and reducing the risk of tumor contact and bleeding. Dexmedetomidine’s mild respiratory depressant effect makes it ideal for awake intubation requiring spontaneous respiration (18). Its high selectivity for α_2_-adrenergic receptors inhibits sympathetic nervous system activity, and this chosen dosing regimen has been shown to reduce intubation-related sympathetic activation (HR increase <10 bpm) in elderly hypertensive patients. This aligns with our patient’s stable intraoperative hemodynamics (BP 148–156/62–68 mmHg, HR 72–80 bpm). Compared to remifentanil (a common alternative), dexmedetomidine avoids respiratory depression (respiratory rate >14 breaths/min in our case) and opioid-related nausea, which is critical for patients at risk of aspiration due to oropharyngeal obstruction.

Effective airway management extends beyond successful intubation to include safe extubation and favorable long-term outcomes. In this case, the endotracheal tube was removed on postoperative day one based on: (i) preoperative imaging confirming no stenosis in the mid- or lower airway and no intraoperative laryngeal injury; (ii) normal postoperative blood gas values (PaO_2_ 95 mmHg, PaCO_2_ 38 mmHg); and (iii) the patient’s alertness and restored swallowing reflex. Extubation was performed promptly to prevent complications associated with prolonged intubation. Histopathology confirmed pleomorphic adenoma with regional malignant transformation (myoepithelial carcinoma), and six-month follow-up showed no recurrence.

This report reflects a single-center, single-case experience. Broader conclusions require validation through larger clinical datasets. Additionally, advanced virtual navigation technologies for 3D airway modeling—such as 3D-printed airway replicas—were not utilized. Incorporation of such technologies may enhance intubation precision in future cases.

For complex airway scenarios involving giant palatal tumors, multimodal imaging—including CT, MRI, and fiberoptic laryngoscopy—is indispensable for informed decision-making. This approach prevents reliance on empirical tracheotomy or blind intubation. Awake transnasal fiberoptic bronchoscope intubation should not be regarded as a high-complexity procedure, but rather as a high-preparation intervention. Key elements for success include comprehensive topical anesthesia, judicious sedative selection, and a robust emergency protocol.

Successful airway management also requires MDT collaboration: surgical teams provide insights into tumor resectability, radiologists clarify anatomical relationships, and anesthesiologists integrate these data into a personalized airway plan.

In conclusion, this case demonstrates that multimodal imaging-guided awake transnasal fiberoptic intubation successfully addressed the airway challenges in an 80-year-old ASA Grade IV patient with a giant palatal pleomorphic adenoma. By avoiding invasive tracheotomy and leveraging evidence-based sedation and anesthesia techniques, this approach achieved optimal safety and patient outcomes, providing a practical framework for managing similarly complex cases.

## 4 Patient’s perspective

We invited the patient to share his reflections on the awake intubation process and postoperative recovery. This is his account: “I’m deeply grateful to the entire medical team for their meticulous care. Before the procedure, the anesthesiologists explained the awake transnasal intubation plan in detail, which really eased my anxiety about being awake during the process. During intubation, I felt only mild nasal discomfort, and their calm guidance help me stay relaxed. After surgery, I had no hoarseness or sore throat. I’m so grateful they avoided a tracheotomy; I’ve heard friends talk about how tough that recovery can be. I was walking around the ward within days. This experience made me feel truly cared for and safe.”

## Data Availability

The original contributions presented in the study are included in the article/supplementary material. Further inquiries can be directed to the corresponding author.
